# The world’s oldest cerapodan ornithischian dinosaur from the Middle Jurassic of Morocco

**DOI:** 10.1098/rsos.241624

**Published:** 2025-03-12

**Authors:** Susannah Maidment, Driss Ouarhache, Richard J Butler, Khadija Boumir, Ahmed Oussou, Kawtar Ech-charay, Abdessalam El Khanchoufi, Paul M Barrett

**Affiliations:** ^1^ Natural History Museum, London, UK; ^2^ Earth and Environmental Sciences, University of Birmingham, Birmingham, UK; ^3^ Sidi Mohamed Ben Abdellah University, Fes, Morocco

**Keywords:** Bathonian, Ornithopoda, Marginocephalia, femur

## Abstract

The cerapodan dinosaurs were an ornithischian clade that achieved a global distribution in the Cretaceous Period. The ichnological record suggests that these dinosaurs had evolved by the Middle Jurassic, but only a single cerapodan body fossil, an isolated femur from the Callovian of the UK, is known from this interval. In order to elucidate the early stages of cerapodan evolution and help to resolve the many phylogenetic inconsistencies in the clade, new specimens, particularly from historically undersampled localities, are needed. Herein, we report the proximal femur of a cerapodan dinosaur from the Bathonian El Mers III Formation of the Middle Atlas Mountains, Morocco. The specimen, although fragmentary, bears characteristics, including a femoral head offset on a distinct neck and a constriction between the head and greater trochanter, that unite it with Cerapoda to the exclusion of other neornithischians. This specimen represents the world’s oldest cerapodan. The El Mers III Formation has also yielded the world’s oldest ankylosaur (and the first discovered in Africa), as well as one of the oldest stegosaurs. Further sampling of these rocks will therefore be crucial for understanding the radiation of ornithischian dinosaurs.

## 1. Introduction

Cerapoda is a diverse clade of ornithischian dinosaurs with a global distribution. A major component of Cretaceous terrestrial ecosystems, early diverging cerapodans were bipedal, with forelimbs modified for grasping, but by the Late Cretaceous, hadrosaurids and ceratopsids had evolved obligate quadrupedality and sophisticated chewing mechanisms, and they became the dominant herbivores of the Northern Hemisphere [1–5].

Cerapoda is composed of two major clades: Ornithopoda, which includes the non-hadrosaurid iguanodontians and the duck-billed hadrosaurids, and Marginocephalia, which includes the horned, frilled ceratopsians and the dome-headed pachycephalosaurs [6–10]. Although the sister-taxon relationship between these two clades has not been questioned since the advent of cladistic analysis, a great deal of debate remains about cerapodan phylogenetic relationships. A series of small, bipedal ornithischians traditionally known as ‘hypsilophodontids’ are generally recovered as early diverging ornithopods [7,9–11], but Boyd [8] recovered several of them in a clade that formed the sister taxon of Cerapoda. The difference between these two topologies centres on the scoring of just a few characters related to the femur and dentition [12], and we consider these ‘hypsilophodontid’ taxa to be within Cerapoda, following most recent analyses (e.g. [11]). The enigmatic heterodontosaurids, whose members include some of the earliest-known ornithischians, were found as the basalmost clade of Ornithischia by most recent phylogenetic analyses [7–9] but they have also been recovered as a paraphyletic assemblage of early diverging pachycephalosaurs [10], and the idea that heterodontosaurids are related to marginocephalians [13] or lie elsewhere within Cerapoda [6] is not new. The presence of a Gondwanan clade of cerapodans, Elasmaria, that lies either within Ornithopoda [14] or outside of it [8] has also been posited, although the exact membership of that clade varies between analyses.

Cerapodans are well known from the Cretaceous Period, but their Jurassic record is much poorer. Several track sites (e.g. [15–17]) from the Middle Jurassic suggest that large-bodied ornithopods (probably iguanodontians) had already evolved by this time, but their body fossils remain elusive. The earliest definitive cerapodan body fossil is an iguanodontian femur, *Callovosaurus leedsi* [18], from the Callovian (Middle Jurassic) Oxford Clay Formation of the UK. The neornithischian *Kulindadromeus*, found in Siberia and preserving bristle-like integumentary structures [19], is of Bathonian age [20], and was recovered within Cerapoda by Dieudonné *et al*. [10], but has generally been considered as a non-cerapodan neornithischian [19,20]. More detailed descriptions and new specimens of this taxon are needed to adequately resolve its phylogenetic position. *Ferganacephale,* from the Bathonian Balabansai Formation of Kyrgyzstan, was described as a pachycephalosaur [21], but it is based on a few water-worn teeth, and its pachycephalosaurian status has been questioned [22,23]. Consequently, the oldest marginocephalian is currently *Yinlong*, from the Oxfordian Shishugou Formation of China [9,13]. To elucidate the early stages of the evolution of Cerapoda and to help resolve the numerous phylogenetic inconsistencies among different analyses, new specimens are needed, especially from historically undersampled localities.

The El Mers III Formation of the Middle Atlas Mountains, Morocco, was deposited on floodplains during the Bathonian [24,25]. The variegated green and red mudstones of the formation are extremely fossiliferous and have so far yielded the remains of the world’s oldest, and first African, ankylosaur, *Spicomellus afer* [26], and one of the oldest stegosaurs, *Adratiklit boulahfa* [27]. Here, we describe a fragmentary but definitive cerapodan from the El Mers III Formation. The specimen, the proximal end of a left femur, is the world’s oldest cerapodan and only the second recorded from the Middle Jurassic globally. The fragmentary nature of the material precludes naming it, but our ongoing work in the El Mers III Formation will hopefully reveal more complete specimens of this taxon in the future.

**Institutional abbreviations**—**CMN**, Canadian Museum of Nature, Ottawa, Canada; **IVPP**, Institute of Vertebrate Palaeontology and Palaeoanthropology, Beijing, PR China; **MNA**, Museum of Northern Arizona, Flagstaff, USA; **NHMUK**, Natural History Museum, London, UK; **OUMNH**, Oxford University Museum of Natural History, Oxford, UK; **USMBA**, Université Sidi Mohamed Ben Abdellah, Palaeontology Collection, Fes, Morocco.

## 2. Material

### 2.1. Specimen

USMBA 001, the proximal portion of a left femur, preserving the femoral head, greater trochanter and proximal-most part of the shaft. Measurements can be found in table 1.

**Table 1.** Measurements of USMBA 001.

measurement	value (mm)
mediolateral width of proximal end of femur, including head	54.4
mediolateral width of shaft	34.7
anteroposterior width of shaft	27.9
maximum anteroposterior width of greater trochanter	32.3
maximum anteroposterior width of head	21.7

### 2.2. Locality and horizon

Bathonian (Middle Jurassic) El Mers III Formation at Boulahfa, near Boulemane, Middle Atlas Mountains, Morocco. The specimen was surface-collected during an expedition to this region by our team in 2020. For more information about the geological setting of the El Mers III Formation at this locality, see Maidment *et al.* [27] for a stratigraphic log of the section, Maidment et al. [26] for further comments on the stratigraphy, and Charrière *et al.* [24] and Oukassou *et al.* [28,29] for dating.

## 3. Description

**Figure 1.**
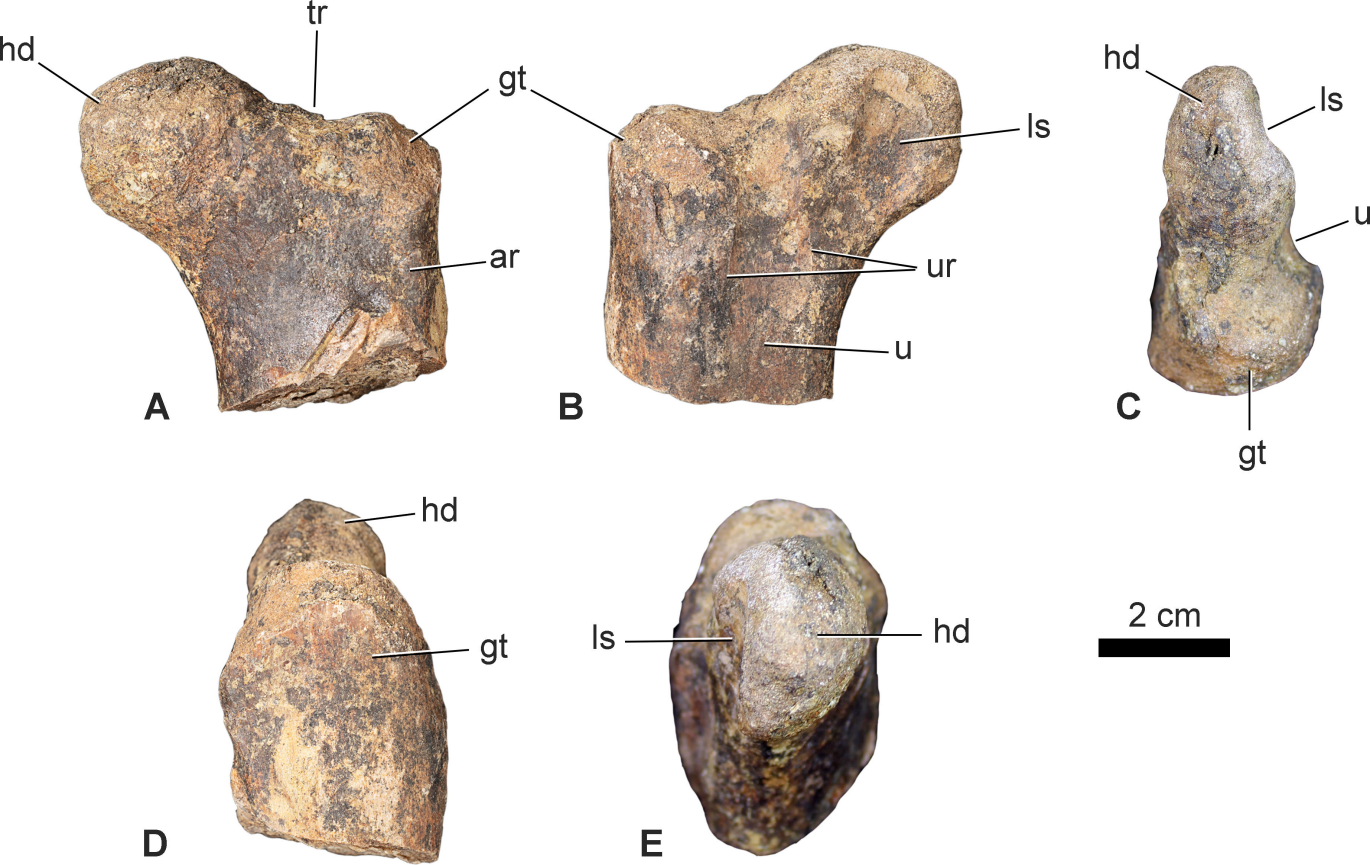
USMBA 001, proximal left femur of a cerapodan dinosaur in A, anterior; B, posterior; C, proximal (dorsal); D, lateral and E, medial views. ar, ridge on the anterior surface; gt, greater trochanter; hd, head; ls, ligament sulcus; tr, trough separating head from greater trochanter; u, u-shaped sulcus; ur, ridges bordering u-shaped sulcus. Scale bar equal to 2 cm.

In anterior view, the femoral head (figure 1A,1, hd) projects dorsomedially from the shaft, and the surface connecting the medial shaft margin and ventral surface of the head is a gentle, continuous concave curve, offsetting the head from the shaft on a distinct neck. A distinct neck between the femoral head and shaft is not present in the earliest-diverging ornithischians and non-cerapodan neornithischians (e.g. *Lesothosaurus*, NHMUK PV RUB 17 [30,31] *Laquintasaura*, NHMUK PV R 36796 [32,33], *Hexinlusaurus* [34]; *Sanxiasaurus* [35] or in thyreophorans (e.g. *Scutellosaurus*, MNA V.175 [36], *Scelidosaurus*, NHMUK PV R 6704 [37]), but is present in all cerapodans [8] (e.g. *Hypsilophodon, Parksosaurus, Haya* and *Minimicursor;* [37–40]). The dorsal and medial margins of the head are rounded in anterior view, although the medial margin is slightly eroded and thus appears somewhat flattened. A genuinely flattened ventromedial margin of the femoral head, often marked ventrally by a distinct ridge, is present in early diverging ankylopollexian iguanodontians, including *Cumnoria* (OUMNH J3303 [17]), *Camptosaurus*, *Uteodon* [41,42] and *Mantellisaurus* (NHMUK PV R 5764; NHMUK PV R 11521 [43]) and is also present in *Callovosaurus leedsi* (NHMUK PV R 1993). As this feature is absent in dryosaurids (e.g. *Valdosaurus*, NHMUK PV R 184, 185 [44], *Dysalotosaurus*, NHMUK PV R 6861), its presence in *Callovosaurus* calls into question the dryosaurid identification of the specimen by Ruiz-Omeñaca *et al.* [18]. The head is separated from the greater trochanter by a trough-like depression (the trochanteric fossa or fossa trochanteris; figure 1A, tr), a feature which is absent in early diverging ornithischians and non-cerapodan neornithischians (e.g. *Lesothosaurus, Hexinlusaurus, Laquintasaura, Sanxiasaurus* [30,32,34,35]), but present in all cerapodans ([7,9,10]; e.g. *Hypsilophodon, Parksosaurus, Haya* and *Minimicursor*; [38–40,45]). Although the dorsal surface of the greater trochanter (figure 1A, gt) is eroded, it appears unlikely that the greater trochanter would have reached the same height as the dorsal surface of the head in anterior view. This, and the gentle curvature of the head’s ventral margin, gives the head the appearance of projecting strongly dorsomedially when viewed in anterior or posterior view (figure 1A,B). The combination of a dorsomedially projecting femoral head with the low greater trochanter is an unusual feature within Neornithischia, as, in general, the greater trochanter projects to the same level as the head. However, a similar combination of characters is observed in the neoceratopsians *Montanaceratops* [46], *Auroraceratops* (E Morschhauser 2023, personal communication), *Archaeoceratops* (IVPP V 11115) and the early diverging cerapodan *Thescelosaurus* (NHMUK PV R 3675 [47]). The anterior surface of the shaft is convex, and an irregular ridge (figure 1A, ar) is present extending from the anterior surface of the greater trochanter ventrally. The anterior trochanter is not evident, although it is likely that it was present as a distinct, finger-like process that arose ventral to the preserved portion of the femur and has not been preserved. This indicates that the lesser and greater trochanters were unfused and separated by a deep cleft, and also that the lesser trochanter must have arisen from a relatively distal position on the proximal femur. Fusion of the greater and lesser trochanters is variable in many ornithischians and may be related to ontogenetic stage in some taxa (S.M., R.J.B. and P.M.B., personal observation), but the distally located position of the base of the lesser trochanter is unusual.

In proximal view (figure 1C), the anterior surfaces of the femoral head and shaft are more or less flat: the head does not extend far anteriorly, although it is slightly abraded. However, there is still a clear constriction between the head and greater trochanter (figure 1C), a feature that is absent in non-cerapodan neornithischians (e.g. *Hexinlusaurus*; [7,9]). The posterior surface of the head bears a distinct, ‘U’-shaped ligament sulcus in proximal view (figure 1C, ls). More laterally, the head is distinctly separated from the greater trochanter by a second, more pronounced ‘U’-shaped depression (figure 1C, u). The lateral margin of the greater trochanter is laterally convex and abraded dorsally.

In posterior view, the ligament sulcus (figure 1B, ls) occupies most of the posterior surface of the femoral head and is broad and shallow. It extends from the dorsal margin of the head posteroventrally towards the shaft and merges into the medial margin of the shaft ventrally. This feature is widespread in early diverging cerapodans (e.g. *Jeholosaurus*, IVPP V15939 [48,49]; *Hypsilophodon*, NHMUK PV R 193 [38]; *Thescelosaurus*, NHMUK PV R 3675 [47]), dryosaurids (e.g. *Valdosaurus*, NHMUK PV R 184−185 [44]; *Dysalotosaurus*, NHMUK PV R 6861 [50]), early diverging ankylopollexians (e.g. *Cumnoria*, OUMNH J3303, [51]; *Camptosaurus*, *Uteodon Callovosaurus*, NHMUK PV R 1993 [17,41]) and early diverging neoceratopsians (e.g. *Auroraceratops* [52]; *Leptoceratops*, CMN 8889) but is absent in non-cerapodan ornithischians such as *Hexinlusaurus* and *Lesothosaurus* [30,34] and in hadrosauroids (e.g. *Mantellisaurus*, NHMUK PV R 5674 [43]). The ‘U’-shaped depression separating the head and greater trochanter in dorsal view continues onto the posterior surface of the shaft as a shallow sulcus (figure 1) that extends ventrally, bordered by ridges on either side (figure 1B, ur). This second sulcus is absent in early diverging cerapodans (e.g. *Hypsilophodon*, NHMUK PV R 193 [38]; *Thescelosaurus*, NHMUK PV R 3675), *Cumnoria* (OUMNH J3303 [17] and *Callovosaurus* (NHMUK PV R 1993), but is present to a degree in dryosaurids (e.g. *Valdosaurus*, NHMUK PV R 184−185 [44]; *Dysalotosaurus*, NHMUK PV R 6861) and perhaps *Auroraceratops* [52], E Morschhauser 2023, personal communication) and *Leptoceratops* (CMN 8889) although it is not as well defined as it is in USMBA 001 and it is not bordered by distinct ridges in any of these taxa. This sulcus, bordered by ridges, appears to be unique to the specimen and could be an autapomorphy, but due to the incomplete nature of the material and its uncertain systematic position, we refrain from naming the taxon here. The posterolateral surface of the greater trochanter is strongly convex, while its lateral surface is more gently convex.

## 4. Discussion

### 4.1. Systematic position of USMBA 001

USMBA 001 is a cerapodan because the femoral head is separated from the shaft by a distinct neck, a feature that is absent in early diverging ornithischians, non-cerapodan neornithischians and thyreophorans (see above [7,8]). The specimen is not a non-cerapodan neornithischian because there is a clear constriction between the head and greater trochanter (a trochanteric fossa), a feature absent in those taxa (see above [7,9]). The presence of a ligament sulcus on the posterior surface of the femoral head indicates that the specimen is an early diverging cerapodan, early diverging iguanodontian or early diverging ceratopsian, but excludes it from being a non-cerapodan neornithischian, hadrosauroid or ceratopsid because the ligament sulcus was not present in these taxa (see description, above). The lack of a ventromedially flattened femoral head flanked ventrally by a distinct ridge excludes USMBA 001 from Ankylopollexia (the least inclusive clade containing *Camptosaurus dispar*, *Uteodon aphanoecetes* and *Parasaurolophus walkeri* [53]). The dorsomedially projecting head is seen in some early diverging ceratopsians such as *Archaeoceratops* (IVPP V 11115), *Auroraceratops* (E Morschhauser 2023, personal communication) and *Montanaceratops* [46], but it is also present in *Thescelosaurus* (NHMUK PV R 3675). USMBA 001 therefore shares features in common with a range of early diverging cerapodan ornithischians but is clearly not a non-cerapodan neornithischian. However, until more complete material is found, we are unable to more accurately determine its systematic position.

### 4.2. The Middle Jurassic as the cradle of ornithischian innovation

Prior to the Middle Jurassic, ornithischian dinosaurs were generally small and bipedal (with the exception of *Scelidosaurus* and *Yuxisaurus*, which are armoured thyreophorans and the first ornithischians to evolve quadrupedality [54–56]). These taxa formed a relatively minor component of Early Jurassic terrestrial ecosystems and were outweighed both in abundance and body size by the early sauropodomorph dinosaurs, a paraphyletic assemblage of herbivores that radiated in the Late Triassic and dominated the terrestrial herbivorous niche until the Middle Jurassic [57].

The Middle Jurassic, however, witnessed evolutionary innovations and body size increases across the ornithischian phylogenetic tree, with the origins and radiations of stegosaurs, ankylosaurs and cerapodans. The Toarcian Oceanic Anoxic Event, also known as the Jenkyns Event [58] occurred towards the end of the Early Jurassic and is marked by a significant negative δ^13^C excursion correlated with extremely high sea surface temperatures, increases in chemical weathering and high sea levels, which have all been linked with the eruption of the Karoo-Ferrar Large Igneous Province [59]. Although the effects of the Jenkyns Event on land are less well studied than in the marine realm, the climate is expected to have become warmer and more humid than before or after [60]. Intervals of climatic upheaval are frequently considered responsible for major biotic turnovers, and it has been suggested that the Jenkyns Event might have caused the extinction of non-sauropod sauropodomorphs, perhaps leaving vacant ecological space into which ornithischians and sauropods could radiate [60]. However, sampling in the Early Jurassic is relatively poor, and studies that take sampling bias into account are needed across the Jenkyns Event to fully test this hypothesis. Nevertheless, the Middle Jurassic appears to have been a critical time in the origin and establishment of dinosaur-dominated terrestrial ecosystems, and further work on this historically undersampled interval will enable us to more clearly elucidate the drivers of evolutionary innovations that allowed ornithischians to radiate and establish themselves as dominant herbivores in the Mesozoic landscape. Further sampling of the El Mers III Formation of Morocco is crucial for understanding the Middle Jurassic radiation of ornithischian dinosaurs, given that limited sampling of the formation to date has already yielded the stratigraphically oldest known ankylosaur, one of the oldest known stegosaurs, and now the oldest known cerapodan.

## Data Availability

This article has no additional data.
